# Analyzing angular momentum in the takeoff phase of medium-hill ski jumping

**DOI:** 10.3389/fspor.2025.1643241

**Published:** 2025-09-01

**Authors:** Yuta Funato, Hirotaka Nakashima, Shinji Sakurai

**Affiliations:** ^1^ Graduate School of Health and Sport Sciences, Chukyo University, Toyota, Japan; ^2^ Department of Sport Science and Research, Japan Institute of Sports Sciences, Tokyo, Japan; ^3^ School of Health and Sport Sciences, Chukyo University, Toyota, Japan

**Keywords:** angular momentum, coaching, ski jumping, medium hill, takeoff phase

## Abstract

**Introduction:**

Athletic performance in competitive ski jumping is evaluated based on the aggregate scores of the jump distance and flying style. However, an understanding of how angular momentum influences performance, particularly during the approach to takeoff phases, is lacking. Therefore, this study aimed to quantify the angular momentum during the takeoff motion on a medium hill and to examine the appropriate angular momentum.

**Methods:**

The study participants included 21 jumpers (16 males and five females; height: 1.65 ± 0.09 m; total weight: 54.6 ± 8.9 kg; age: 19.2 ± 6.8 years) performing on a medium hill. The angular momentum of the center of gravity of the jumper + ski system (AM_CG_) at takeoff was filmed at 200 Hz using a high-speed camera and analyzed in the sagittal plane.

**Results:**

As a result of confirming the relationship between AM_CG_ at takeoff and jump distance, a significant quadratic approximation curve was obtained, indicating that the value at the apex of the *X*-axis was 0.0391 s^−1^ (*p* < 0.05).

**Discussion:**

This study shows that the appropriate AM_CG_ value at takeoff was approximately 0.0391 s^−1^. The study findings are expected to contribute to coaching with objective indicators.

## 1 Introduction

The performance of athletes in competitive ski jumping is evaluated based on the jump distance and flying style, with the aggregate of these points determining their overall score. The significance of jump distance in this scoring system elevates it as a primary determinant of competitive success.

Ski jumping involves a sequence of movements segmented into four phases: the approach, takeoff, flight, and landing. The jump distance is influenced by the initial flight phase conditions and the forces acting on the jumper and the center of gravity (CG) of the ski system during flight. Despite the constrained lift generation of the flight phase, it predominantly comprises falling motion under gravitational influence. As such, the takeoff phase is critical because it sets the initial conditions for the flight phase (1, 2).

Two primary mechanical objectives exist during the takeoff aimed at increasing jump distance, as outlined by Schwameder (1). The first objective is to “attain vertical velocity at the jumper's CG during takeoff.” The second objective is to “generate appropriate forward angular momentum around the jumper's CG.”

Numerous studies have focused on the first objective, and in the actual jump scenario, a positive correlation has been observed between the impulse of the ground reaction force during the takeoff motion and the vertical velocity of the CG of the jumper, as captured by video cameras or the resulting jump distance achieved, respectively (3–6). These findings confirm the importance of the first objective.

Conversely, only a few studies have focused on the second objective. Ettema et al. (7, 8) calculated the angular momentum in simulated jumps that mimicked actual takeoff motion. These simulations included athlete jumps with friction at the foot sole and jumps on a board with rollers, performed on a −2° slope at speeds of approximately 1.6 m/s, where foot sole friction is negligible. However, recognizing that the conditions for simulated jumps differ from those of actual jumps is crucial. In the actual hill jump, the ground reaction force vector only acts vertically to the takeoff table at a speed of approximately 20 m/s. The difference in velocity influences angular momentum, limiting the quantification of angular momentum in simulated jumps.

Schwameder (1) analyzed the angular momentum in actual ski jumping takeoff. Building upon the study by Schwameder and Muller (9), Schwameder (1) calculated the angular momentum around the CG of the jumper during the takeoff motion of a highly skilled athlete. This calculation involved utilizing plantar pressure data obtained from an insole-type pressure meter in conjunction with coordinate data sourced from video camera footage. Schwameder (1) presented the changes as a time series; however, the study did not delve into the magnitude or examine its relationship with jump distance in detail. This gap highlights a lack of comprehensive understanding of how angular momentum influences performance, particularly from the approach to takeoff phases. Previous studies extensively cover the takeoff phase (1, 7, 8), yet there remains a dearth of detailed examination of the angular momentum of the jumper throughout the entire approach and takeoff sequence. Yamanobe (10) further emphasized the need to provide (in advance) angular momentum around the CG of the jumper during the takeoff motion. However, the required angular momentum during takeoff to obtain an appropriate forward tilt posture is unknown. Therefore, quantifying the angular momentum is crucial.

Obtaining the change in angular momentum of the takeoff motion of jumpers with a wide range of skills on the medium hill and examining the appropriate angular momentum can serve as fundamental reference data.

Therefore, this study investigates the approach and takeoff phases using a medium hill scenario. We aim to examine and quantify the appropriate angular momentum during the takeoff motion on the medium hill. The approach phase, spanning 55.2 m from the start gate to takeoff, involves descending a −35° slope, transitioning through a 59 m radius curve, and concluding with a 5.5 m straight takeoff section at a −9.5° angle (takeoff table). During this time, the coefficient of friction between the skis of jumpers and the snow surface is minimal, resulting in a limited frictional force (1). This implies that the takeoff motion must be made in the straight section at −9.5°, where the friction force is approximately zero. This can be described as a situation in which the ground reaction force vector only acts vertically to the takeoff table.

## 2 Methods

The participants and trials leveraged in this study are identical to those by Funato and Sakurai (6). A brief description is provided below. Funato and Sakurai (6) focused on the first mechanical objectives of the takeoff phase, whereas this study focused on the second mechanical objective. Therefore, the two studies are clearly different.

### 2.1 Participants

Twenty-one ski jumpers participated in this study, comprising 16 males and 5 females. Their physical characteristics were as follows: an average height of 1.65 ± 0.09 m, an average body mass of 54.6 ± 8.9 kg, and an average age of 19.2 ± 6.8 years. Notably, the participants encompassed a wide range of jumping proficiency levels, ranging from elite jumpers with Olympic experience to elementary school students.

Ethical approval for this study was obtained from the Ethics Committee of Chukyo University, and informed consent was acquired from all participants. In cases where a participant was a minor, consent was obtained from their parents or legal guardians.

### 2.2 Data collection and smoothing

Ski jumping hills are classified into five levels, following the regulations of the International Ski Federation (FIS): small, medium, normal, large, and flying hills (11). Medium hills are particularly versatile, accommodating jumpers with varying levels of technique.

The study was conducted on a medium summer hill with a hill size of 68.0 m. Throughout the experimental trials, all participants initiated their jumps from a standardized gate position, and their ski-run surfaces were treated with paraffin wax. Although wind measurements were unavailable, the conditions were characterized by minimal wind interference, ranging from nearly windless to light winds, maintaining relatively consistent conditions. On the day of the experiment, the average wind speed in the area was 2.2 m/s, as reported by the Japan Meteorological Agency. The average value of the component in the movement direction of the jumper was a headwind of 0.6 m/s.

Two synchronized high-speed digital video cameras (Phantom Miro 4c, Vision Research Inc.) operating at 200 Hz with a shutter speed of 1/1,000 were employed to capture the takeoff motions of the jumpers. These cameras utilize 3D direct linear transformation filming and calibration techniques.

The jump distance was measured using a digital video camera (HDR-CX700, SONY) positioned alongside the landing area, adhering to the regulations outlined by the FIS, i.e., “The landing is considered complete when both feet are in full contact with the landing slope. For abnormal landings (one-foot landing, e.g., one foot on the snow and the other remaining in the air noticeably longer than it would take for a normal landing), the jump distance measured will be the point where the first foot is in full contact with the landing slope.”

Participants were instructed to perform the jump task one to three times, and for those who executed multiple jumps, the attempt yielding the greatest jump distance was chosen for subsequent analysis.

A stationary coordinate system was established for the analysis, with the *x*-axis horizontal to the direction of progression of the jumper, the *y*-axis oriented vertically upward, and the z-axis representing the left–right directions. The average standard errors in the real coordinates were 0.007 m, 0.010 m, and 0.011 m in the X, Y, and Z directions, respectively. These errors correspond to 0.11%, 0.50%, and 1.38% within a calibration range of 6.1 m, 2.0 m, and 0.8 m, respectively.

From the video data, 11 key points located on the left side of the body were manually digitized using motion analysis software (Frame-DIAS V, DKH) and smoothed using a Butterworth low pass filter, with a cutoff frequency of 8 Hz. These points included the vertex, tragus, shoulder, elbow, wrist, top of the finger, hip joint, knee joint, ankle, heel, and toe.

The ski specifications of each jumper were determined based on the data derived from one participant. First, the ski mass was identified as 6.405 kg. Subsequently, using the center of mass board method, the center of mass of the ski was pinpointed at 57.3643% from the tip. Next, the height and weight of the jumper was to determine the length of the ski (e.g., for a BMI of 21 or higher, a ski length equal to 145% of the height of the jumper was applied). Consequently, the ski length of each participant was ascertained using these data. Furthermore, the ski mass for each participant was estimated referencing the ski mass obtained from one participant. According to the FIS rule, the toes are fixed at 57% from the tip of the skis. Therefore, the CG of the skis was determined to be parallel to the takeoff table based on the toes. The ski segment of each participant was constructed based on the calculated center of mass.

**Figure 1:**
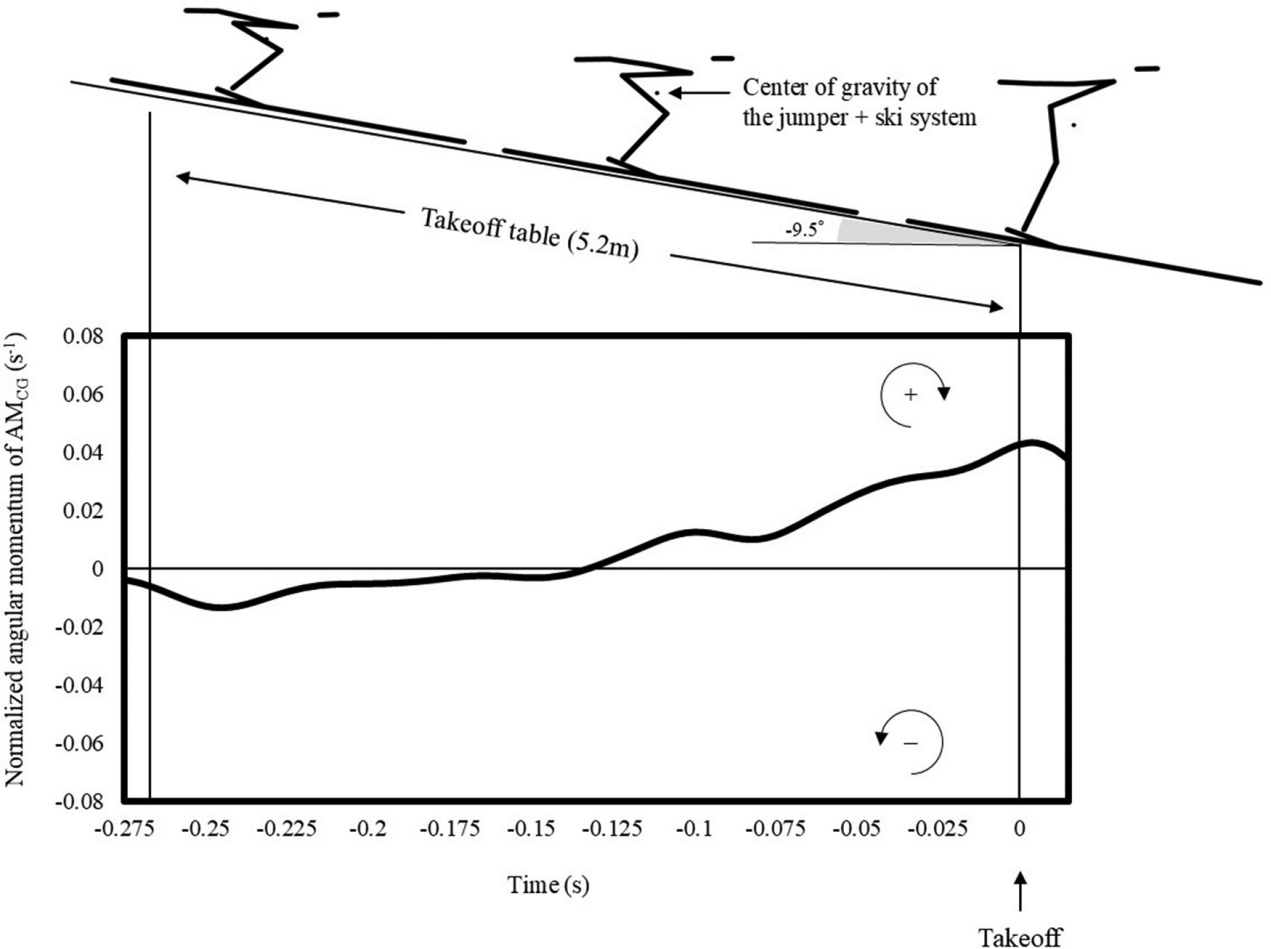
An example of changes in angular momentum.

The motion of ski jumping is generally considered to be symmetrical. Computer simulation models are also modeled symmetrically [e.g., (12)]. Therefore, a nine-segment model was then constructed for the jumper + ski and subsequently analyzed in the sagittal plane (Figure 1).

### 2.3 Calculated items

#### 2.3.1 Angular momentum

The angular momentum of each body part ($H_{i}$) was calculated using the equation defined by Dapena (13) and Hay et al. (14)

$$H_{i}=r_{i/G}\;\times m_{i}V_{i}+I_{i}\omega_{i}$$

where $r_{i/G}$ represents the position vector of the CG of body part *i* ($CG_{i}$) relative to the CG of the jumper + ski system*,* $m_{i}$ denotes the mass of *i*, $V_{i}$ denotes the velocity vector of the $CG_{i}$ relative to the CG of the jumper + ski system, $I_{i}$ denotes the moment of inertia of body part *i* about its partial CG, and $\omega_{i}$ is the angular velocity vector of body part *i*. Notably, the inertial properties of the body parts were estimated using the inertial parameters of the body segments proposed by Ae (15) and Yokoi et al. (16). The equation is divided into two parts: the first term on the right side accounts for the angular momentum of the CG of body part i relative to the overall CG of the jumper + ski system, often referred to as the “transfer term.” The second term deals with the angular momentum of body part *i* around its own CG, commonly referred to as the “local term.”

The takeoff table is a flat surface, as the jump stands are made according to the profile specified in the rules. The skis thus have no angular velocity on the takeoff table. Therefore, only the transfer term is calculated for the angular momentum of the skis, and the local term is assumed to be zero. The same conditions apply to snow since the grooves are created by ice.

To determine the angular momentum of the CG of the jumper + ski system (AM_CG_), the angular momenta of all body parts were summed together using the following equation:

$$AM_{CG}=\overset{9}{\underset{i=1}{\sum }}\;H_{i}\;$$

The body was segmented into the head-trunk (AM_Head–trunk_, encompassing head and trunk), arms (AM_Arms_, including the upper arm, forearm, and hand), and legs–ski (AM_Legs–ski_, comprising thigh, lower leg, foot, and ski), to further understand which body parts contribute to generating angular momentum following Hinrichs (17). Subsequently, the angular momentum for each section was calculated.

Standardization of the calculated angular momentum was achieved by dividing it by the product of the square of the height and total weight (body and ski masses) of each jumper, a method also outlined by Hinrichs (17). The unit for this standardized angular momentum is expressed as s^−1^. In this study, a forward rotation is assigned a positive value, while a backward rotation is considered negative.

### 2.4 Statistics

The relationship between AM_CG_ at takeoff and jump distance was confirmed using curve regression analysis. The significance level for statistical analysis was set to 5%.

## 3 Results

**Figure 2:**
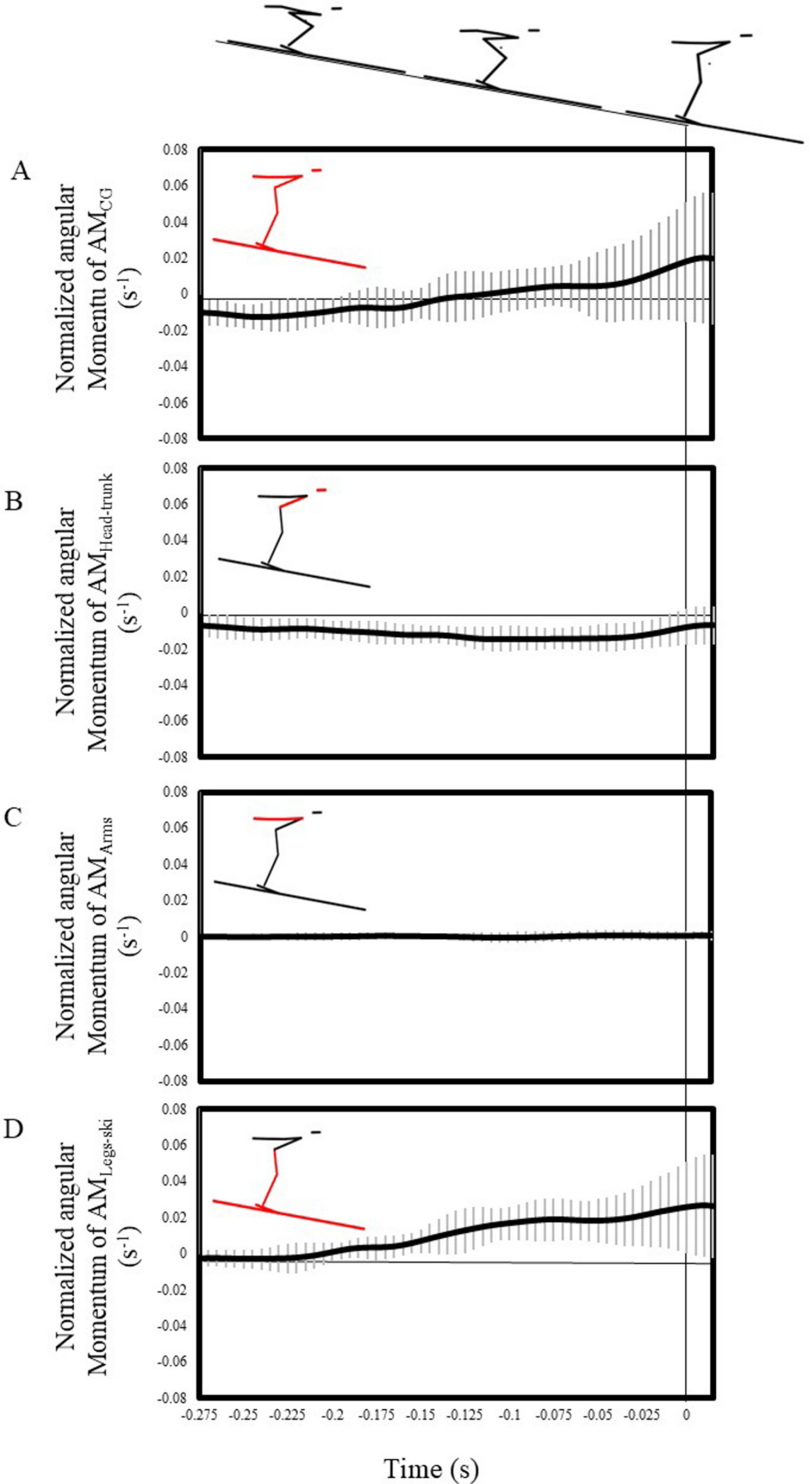
The average values of AM_CG_ **(A)**, AM_Head–trunk_ **(B)**, AM_Arms_ **(C)**, and AM_Legs–ski_ **(D)** over time. The black line represents the average value, and the gray line represents the standard deviation.

Figure 2 shows the time series of AM_CG_ and the angular momentum of each body part. The AM_CG_ was approximately −0.01 s^−1^ in the first half of the takeoff motion; however, its value became positive in the middle of the takeoff motion. The value at takeoff was 0.018 ± 0.035 s^−1^ (Figure 2A). The AM_Head–trunk_ value was approximately −0.01 s^−1^ from beginning to end (Figure 2B). AM_Arms_ exhibited a value of approximately zero throughout (Figure 2C). Meanwhile, AM_Legs–ski_ had a value of approximately 0 s^−^¹ in the first half of the takeoff motion, shifted to a positive value in the latter half, and exhibited a positive value at takeoff (Figure 2D).

**Figure 3:**
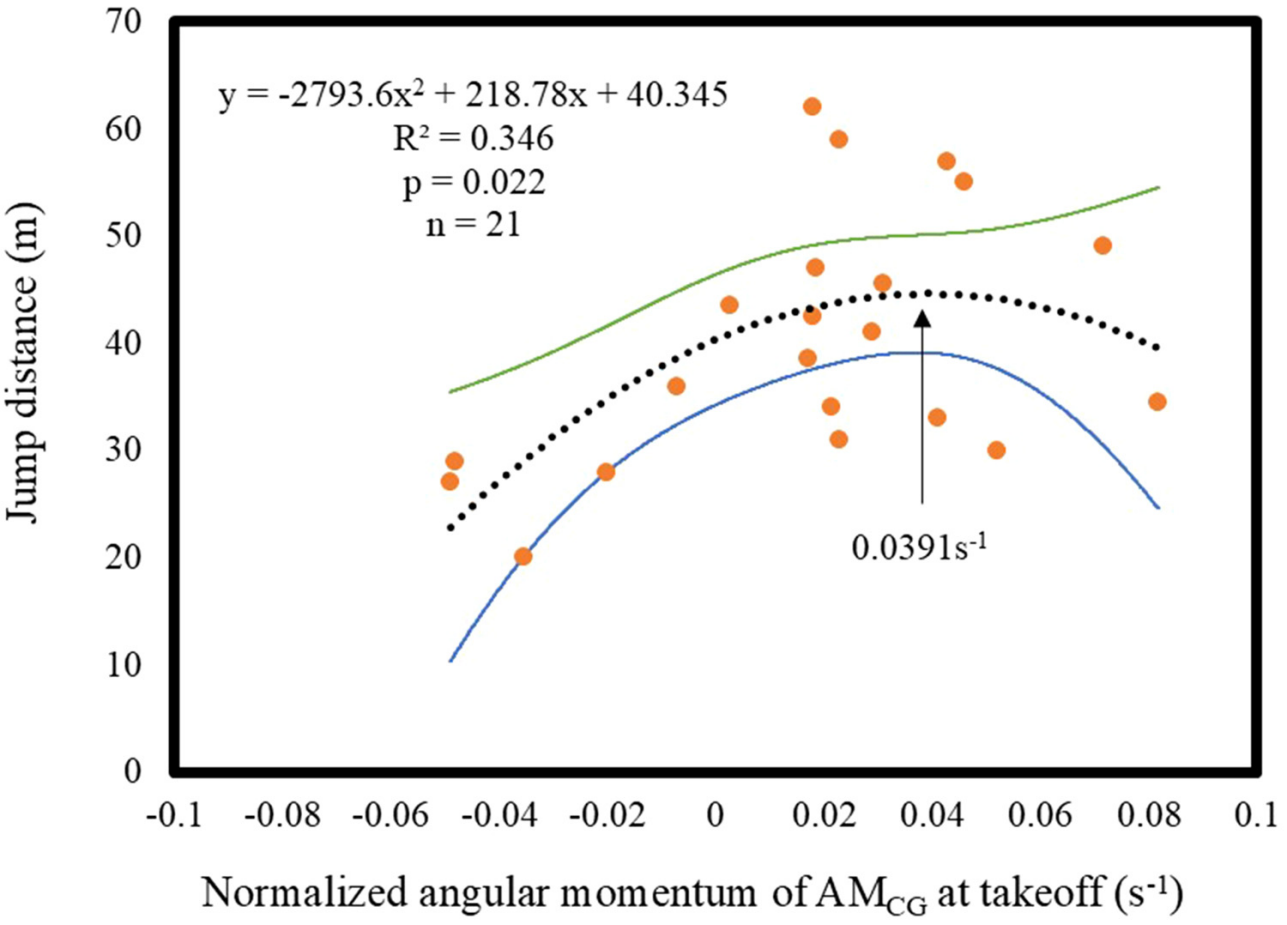
Relationship between jump distance and the AM_CG_ at takeoff. The orange dots represent the data points. The black dashed line represents the fitted curve. The green line represents the upper bound of the 95% confidence interval, and the blue line represents the lower bound.

Figure 3 shows the relationship between AM_CG_ at takeoff and the jump distance. The results of the curve regression analysis revealed a significant quadratic function approximation curve, indicating that the value of the apex of the *X*-axis was 0.0391 s^−1^ (*p* < 0.05). The *R*^2^ value was 0.346.

## 4 Discussion

To the best of our knowledge, this is the first study investigating the angular momentum during ski jumping, which, despite being considered important during the takeoff motion, had previously remained unknown. Main findings of this study are two-fold; First, an increase in AM_CG_ does not necessarily lead to an increase in the jump distance. Second, the appropriate value of AM_CG_ at takeoff was found to be approximately 0.0391 s^−^¹. The details are discussed below.

### 4.1 Relationship between angular momentum of the center of gravity of the jumper + ski system and jump distance

As shown in Figure 2A, the AM_CG_ exhibited a slightly negative angular momentum in the first half of the takeoff motion, similar to Schwameder (1). During actual jumps, air resistance imparts a backward moment. Wind tunnel experiments on takeoff motions conducted by Virmavirta et al. (18) indicated a notable increase in the angle between the trunk and the ground due to air resistance. This suggests that the aerodynamic force must have recorded backward AM_CG_ during the first half of the takeoff motion.

In the middle phase of the takeoff motion, the AM_CG_ changed to a positive value. Consequently, the jumpers obtained a forward AM_CG_ at takeoff. However, as shown in Figure 3, the relationship between AM_CG_ and the jump distance at takeoff was not linear, indicating that an increase in AM_CG_ does not necessarily lead to an increase in jump distance. This finding was also consistent with that by Schwameder (1), who describe the objective “generate appropriate forward angular momentum around the jumper's CG.”

As shown in Figure 3, the X value of the apex of the approximation curve was 0.0391 s^−^¹. This result indicates that the appropriate AM_CG_ value at takeoff is approximately 0.0391 s^−^¹. In addition, in Figure 3, a large variation from the approximate curve was observed, and the coefficient of determination was not high. This may be because not only the angular momentum, but also the initial velocity, and the angle of the velocity vector at takeoff affect the determination of the jump distance. A sample size of 21 jumpers was used in this study. Therefore, a *post hoc* test was performed. The results showed that the power (1-β) was 0.88.

The AM_CG_ at takeoff and the jump distance were related by a quadratic function curve for two possible reasons. First, excessive forward AM_CG_ may cause a fall. Second, the generation of AM_CG_ may affect the vertical velocity of the jumper's CG. The AM_CG_ generated by the jumper is determined by the length between the ground reaction force vector (GRFV) and the jumper's CG. The GRFV can be divided into the vector acting on the jumper's CG and the vector generating the AM_CG_. Furthermore, the vector acting on the jumper's CG can be divided into components acting in the vertical and horizontal directions. Assuming a fixed GRFV, the longer the length between the GRFV and the jumper's CG (moment arm), the larger the AM_CG_. However, the vertical component of the vector acting on the jumper's CG becomes smaller. Consequently, it can be considered that, although the center of pressure of the GRFV is unknown in this study, when AM_CG_ becomes too large, the vertical velocity of the jumper's CG cannot be sufficiently obtained, resulting in a shorter jump distance.

One purpose for obtaining an appropriate AM_CG_ is to smoothly transition to the flight position. In fact, Virmavirta et al. (19) reported that the angle between the body and the horizontal axis should be small for 0.5 s immediately after takeoff. In addition, Virmavirta et al. (19) showed in their study on large hills that a significant negative correlation exists between the vertical velocity of the jumper's CG immediately after takeoff and the flight distance. These studies suggest that obtaining an appropriate AM_CG_ is crucial, even at the expense of components that contribute to the vertical velocity of the jumper's CG in the GRFV, particularly in large hills.

### 4.2 Practical implications

Funato and Sakurai (6) indicated the importance of maintaining a low shank angle and a high angular velocity of the knee joint extension during the takeoff motion. This motion thus contributes to the positive angular momentum of the local term of the thigh and the positive angular momentum of the transfer term of the AM_Head–trunk_. In addition, Funato and Sakurai (6) demonstrated the importance of reducing the trunk angle, indicating that this motion contributes to controlling the negative angular momentum of the local term of the AM_Head–trunk_. These specific movements not only contribute to increasing the vertical velocity of the jumper's CG while minimizing the frontal projected area exposed to drag forces but also aid in acquiring forward AM_CG_.

Ski jumping instruction is generally based on the subjectivity of the coach. For example, beginners mainly begin their training on small or medium hills, and as they progress, they increase the size of their hill jumps. The timing of this switch is basically left to the subjectivity of the coach. In particular, in this study, some jumpers recorded negative AM_CG_ values at takeoff. Given that the larger the hill size, the more important it is to have an appropriate forward AM_CG_, it can be considered that at least a positive AM_CG_ value is an indicator for considering a change in the hill size. In particular, for junior jumpers, it is essential to consider that obtaining AM_CG_ may depend on muscle strength.

This study successfully quantified angular momentum values, combining the forward angular momentum produced by the jumper around the CG of the jumper + ski system and the backward angular momentum due to air resistance. Notably, these calculations did not account for air resistance during simulated jumps.

### 4.3 Limitations

The limitation of this study is that direct air resistance could not be measured.

We analyzed the best jumps for jumpers who performed two or more jumps to eliminate jumps in which jumpers who were clearly aware of their mistakes in the takeoff phase broke their flight position voluntarily during the flight phase and did not maximize their jump distance. Among the jumpers who did not make such jumps, the difference in jump distance between the two jumpers with the largest differences was 3 m (participant 1: 62–59 m, participant 2: 20–17 m). Consequently, it can be considered that the jumps used in this study were not outliers and were appropriate. Furthermore, since the confidence interval for the relationship between the AM_CG_ at takeoff and the jump distance is not wide, the accuracy of the estimation is considered to be acceptable (Figure 3).

**Figure 4:**
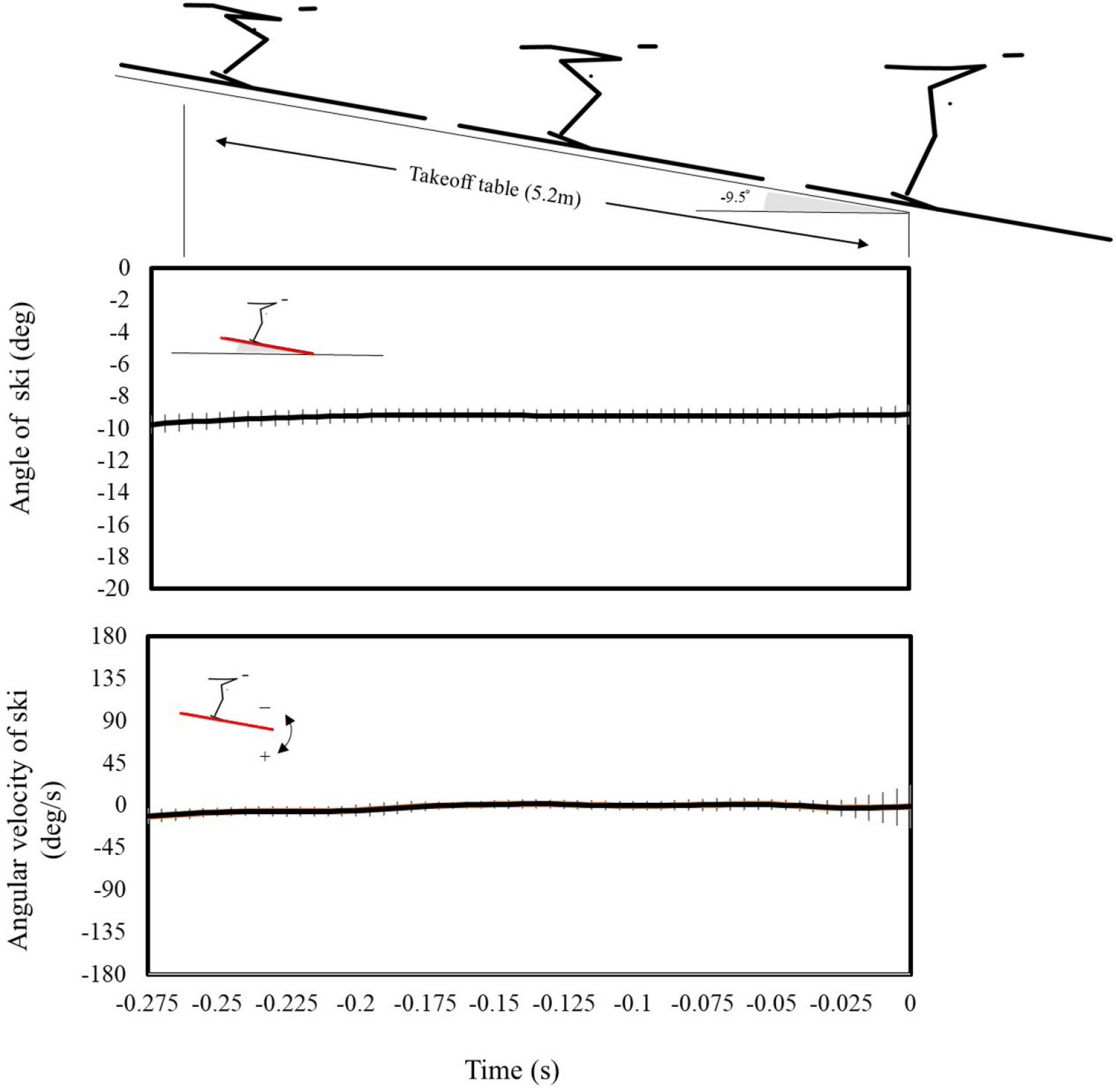
Average values of the ski angle and angular velocity over time. The black line represents the average value, and the gray line represents the standard deviation.

In this study, the local term for skiing was calculated to zero. The angle and angular velocity of the skis were examined (Figure 4) to validate this result. Consequently, the angular velocity of the skis was approximately zero. Note that before approximately −0.27 s, the curve before the takeoff table caused some angular changes. In addition, the slight angular velocity recorded just before takeoff was because the ski tip was slightly bent. Therefore, we determined that calculating the local term of the ski as zero in future AM_CG_ calculations poses no issues.

## 5 Conclusion

We aimed to quantify the angular momentum during the takeoff motion on a medium hill and to examine the appropriate angular momentum. The study participants included 21 jumpers performing on a medium hill. The AM_CG_ at takeoff was filmed at 200 Hz using a high-speed camera and analyzed in the sagittal plane. The results showed that an increase in AM_CG_ does not necessarily lead to an increase in jump distance. In addition, the appropriate value of AM_CG_ at takeoff was found to be approximately 0.0391 s^−^¹. The findings of this study are expected to contribute to coaching with objective indicators.

## Data Availability

The original contributions presented in the study are included in the article/Supplementary Material, further inquiries can be directed to the corresponding author.
